# Role of dietary fatty acids in microglial polarization in Alzheimer’s disease

**DOI:** 10.1186/s12974-020-01742-3

**Published:** 2020-03-24

**Authors:** Smita Eknath Desale, Subashchandrabose Chinnathambi

**Affiliations:** 1 Neurobiology Group, Division of Biochemical Sciences, CSIR-National Chemical Laboratory, Dr. Homi Bhabha Road, Pune, 411008 India; 2 Academy of Scientific and Innovative Research (AcSIR), Pune, 411008 India

**Keywords:** Fatty acids, Omega-3 fatty acids, Tau phagocytosis, Tau inflammation, Tauopathy, Tau spreading, Microglial polarization

## Abstract

Microglial polarization is an utmost important phenomenon in Alzheimer’s disease that influences the brain environment. Polarization depends upon the types of responses that cells undergo, and it is characterized by receptors present on the cell surface and the secreted cytokines to the most. The expression of receptors on the surface is majorly influenced by internal and external factors such as dietary lipids. Types of fatty acids consumed through diet influence the brain environment and glial cell phenotype and types of receptors on microglia. Reports suggest that dietary habits influence microglial polarization and the switching of microglial phenotype is very important in neurodegenerative diseases. Omega-3 fatty acids have more influence on the brain, and they are found to regulate the inflammatory stage of microglia by fine-tuning the number of receptors expressed on microglia cells. In Alzheimer’s disease, one of the pathological proteins involved is Tau protein, and microtubule-associated protein upon abnormal phosphorylation detaches from the microtubule and forms insoluble aggregates. Aggregated proteins have a tendency to propagate within the neurons and also become one of the causes of neuroinflammation. We hypothesize that tuning microglia towards anti-inflammatory phenotype would reduce the propagation of Tau in Alzheimer’s disease.

## Background

Gliosis is a consequence of aberrant activation of microglia and astrocytes that is marked with increased expression of pro-inflammatory cytokines, leading to neuroinflammation. Microglia specifically have two phenotypes: pro-inflammatory and anti-inflammatory; this polarization of microglia depends upon the type of secreted cytokines and markers that they express on the cell surface. This polarization of microglia is influenced by many internal and external factors including dietary fatty acids. Omega-3 fatty acids especially have beneficial effects on the brain and effectively regulate microglial polarization, which is based on the type of lipid mediators they regulate. In this study, we infer that the neuroinflammatory reaction during Alzheimer’s disease could be regulated by dietary intake of fatty acids, which can also enhance microglia polarization towards the anti-inflammatory phase. Tau protein, on the other side, has the ability to aggregate, accumulate in neurons, and also show the ability to propagate within the neuron similar to prion-like diseases. In this study, we hypothesize that polarizing microglia with dietary fatty acids can reduce the propagation of Tau.

## Neurodegeneration and Tauopathies

Progressive neuronal loss is the most commonly known hallmarks of neurodegeneration. The neuronal loss is a consequence of the accumulation of abnormal protein, defects in neurotransmitter receptors, and alteration in signaling pathways, which ultimately leads to progressive loss of structure and function of a neuron, including death of neurons [1, 2]. Progressive neuronal loss affects the number of neuron and neuronal connections and increases complexity in a neuronal network [1, 3]. In neurodegeneration, synaptic loss is primarily spotted due to lost neuronal connections and deposition of aberrant proteins. Neuronal loss majorly leads to dementia, which is a major consequence of Alzheimer’s disease (AD), Parkinson’s disease (PD), Huntington’s disease (HD), and other neurodegenerative diseases. Memory loss and cognitive impairments are the main pathological symptoms along with physiological impairments such as difficulties in speaking and breathing and affecting body movement in the later stages of the disease [4, 5]. Changes in neuronal numbers and loss of neuronal connection are the main reason for fluctuations in behavior. These fluctuations are reversible and can be overcome by the enhancement of neuronal plasticity [6, 7]. The accumulation of aberrant proteins amyloid-β plaques and neurofibrillary tangles of Tau are the main reasons for neuronal network loss in many neurodegenerative diseases. Switching to alternative neuronal connection is the way to overcome this loss, which depends upon the plasticity of the brain [8]. The consistent failure of neuronal connections is responsible for daily fluctuations in behavior, and they are interconnected with the accumulation of plaques and tangles, which in turn cause neuronal loss and network dysfunction [9, 10].

In Alzheimer’s disease, the synaptic loss is prominent due to the accumulation of Aβ protein in synapses, which affect neurotransmission and thus ultimately cause neuronal loss. Tau, a microtubule-associated protein, is a vital component of intracellular neurofibrillary tangles (NFTs) in neurodegenerative diseases specially Tauopathies including AD. In the hyperphosphorylated state, which is predominant in AD, Tau loses its native conformation and accumulates in the cell in the form of misfolded protein. These misfolded, aggregated forms of Tau then bundle up to form NFTs [11, 12]. Excessive accumulation of NFTs causes neuronal death and results in neurodegeneration [12, 13]. Accumulation of Aβ also triggers phosphorylation of Tau leading to NFT formation and enhances Tau-mediated cytotoxicity [14]. Due to hyperphosphorylation, Tau disrupts from microtubule filaments affecting the axonal stability, and Tau bundles up to form NFTs; accumulation of this leads to death of neurons, which prevails neurodegeneration [12, 15, 16]. Along with the accumulation of aberrant proteins, glial cells mainly astrocytes and microglia play a major role in impairing neuronal network. Aberrant activation of glial cells due to the presence of aberrant proteins in the brain environment causes inflammation and neuronal death [10, 17, 18].

## Neuroinflammation mediated by microglia

Microglia is being a resident immune cell of the brain, involved in surveillance and neuroprotection. Microglia have the ability to migrate, sense the environment, and phagocytose cellular debris, accumulated abnormal pathoproteins, and pathogens [19]. Microglia population predominate the brain environment depending upon the phenotype, i.e., M1 or M2. On immune challenge, microglia primarily undergoes classical activation showing inflammatory response and acts as a first line of defense to destroy the invaded pathogens. The classical activation is followed by the initiation of alternative anti-inflammatory response to repair the wound and tissue damage [20, 21]. The resident non-activated microglia occurs in ramified morphology with the long extension; however, on activation, they tend to retract the extensions and become amoeboid. The morphology change is necessary for the further migration of microglia towards the site of lesion or injury. The conventional knowledge of the brain as an immune-privileged site has been eliminated since the discovery of homeostatic microglia that constantly carry out the immune surveillance. Specific receptors such as P2RY12 mediate the homeostatic nature of microglia [22]. The classical activation is primarily followed by IFN-γ, whereas the alternative activation is by IL-4-mediated immune response [23]. Conventionally, the microglia undergoes classical activation bearing pro-inflammatory response and has been termed as M1 microglia and the alternative activation as M2 microglia that possess anti-inflammatory response. The initial M1/M2-derived nomenclature was assessed to purified stimuli given in in vitro conditions [24]. The single-cell RNA-sequence analysis of microglia suggested a converged expression of M1 and M2 markers due to the influence of disease-related inflammatory process. Hence, the recent studies suggest the nomenclature of M1/M2 as “M1-like” or “M2-like phenotype” based on gene expression profile [24, 25]. The nomenclature has been changed since arresting microglia in a particular phenotype is difficult owing to their plastic nature; the cytokine changes in the environment would switch the phenotype of microglia [26].

M2 anti-inflammatory phenotype is classified as M2a, M2b, and M2c [27]. In M2 alternative activation, M2a is related to repair and regeneration, M2b transitional state is involved in immune response, whereas M2c state is involved in neuroprotection and release of anti-inflammatory cytokines [28]. The newly introduced subtype of microglia named as disease-associated microglia (DAM) possess increased phagocytic ability, antigen presentation and induces clearance of aggregated proteins in neurodegeneration. The myeloid lineage-specific protein TREM-2 regulates important checkpoints and gene expression required for the DAM expression [29, 30].

On classical activation, microglia secretes pro-inflammatory cytokine TNF-γ, IL-1β, TNF-α, and IL-6 as well as reactive oxygen species (ROS), nitric oxide (NO), and other immunomodulatory factors causing inflammatory response at the site of injury [17, 31, 32] [33]. Microglia can show alternative response by secreting anti-inflammatory cytokines such as TGF-β, IL-4, and IL-10, helping in tissue remodeling, repair, and increase of phagocytosis [33–36]. However, there is a very thin differential line between pro-inflammatory and anti-inflammatory phenotype of microglia; hence, characterizing the particular phenotypic population in the brain environment is very critical [24]. In AD condition, pro-inflammatory microglia pre-dominates anti-inflammatory, which impairs the healing mechanism and increases the neuroinflammation [17]. Prolonged activation of microglia at the site of the lesion would increase excessive production of pro-inflammatory cytokines, which causes the death of neurons due to inflammation and also increases the accumulation of toxic proteins [37]. This excessive cytokine secretion increases Tau hyperphosphorylation in AD. In AD, the accumulation of abnormal Aβ peptide in synapses activates the microglia and enhances classical activation. The secreted pro-inflammatory cytokines by microglia may act in autocrine or paracrine mode, which also drives astrocytes activation; this cumulatively enhances neuroinflammation. Gliosis imparts synapse loss and hence increases neuronal dysfunction increasing neurodegeneration [37, 38]. Along with the accumulated Aβ, Tau also enhances the activation of inflammatory pathway by inducing inflammasome, IL-1β, and increased expression of NF-κB pathway [39, 40]. In this process, propagation of Tau species is mediated by insoluble aggregated form, which includes oligomers and NFTs [41, 42]. Of interest, oligomeric species of Tau play a vital role in propagation. This seeding effect of Tau is an early indication of Tauopathies and neurodegeneration, and it also has the potency to trigger activation of glia which gives rise to the inflammatory phase [12, 43–45]. The activation of microglia and its inflammatory cytokines again causes phosphorylation of Tau and increases its misfolding [38, 46–49]. Inflammation-induced Tau phosphorylation is a well-known phenomenon, where the pro-inflammatory cytokines secreted especially IL-1β drive phosphorylation of Tau through p-38 MAPK activation [50]. Microglial activation occurs through LPS treatment as studied by marked increase in IL-1β secretion and LPS-induced hyperphosphorylation of Tau by activating CDK-5 (cyclin-dependent kinase) and GSK-3β (glycogen synthase kinase-3β), which are well-known kinases to be involved in hyperphosphorylation of Tau in pathological conditions [46]. Another pro-inflammatory cytokine TNF-α (tumor necrosis factor-α) induces Tau phosphorylation at specific epitope pT231, a pretangle-associated epitope [51], whereas on the secretion of IFN-γ, which is released on viral infection, causes dephosphorylation of Tau occurring at pretangle-related epitopes [52]. All the evidences denote that Tau pathology associated with inflammation depends on the type of pro-inflammatory response.

## Effect of fatty acids on microglia

**Fig. 1:**
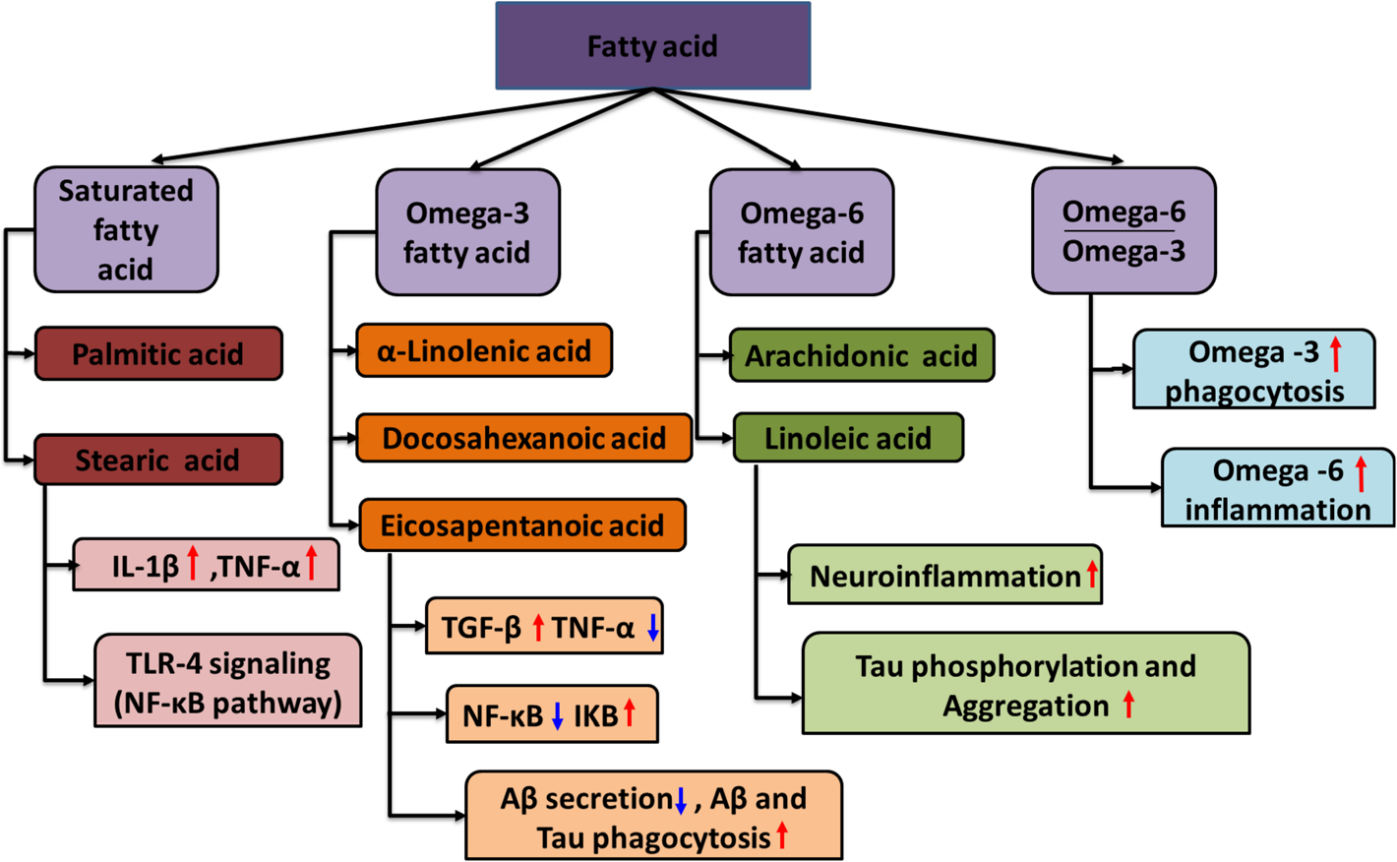
Overview of fatty acids affecting inflammatory and phagocytic pathways. AD is associated with the presence of Aβ, neurofibrillary tangles of Tau protein, and excessive accumulation of glia cells, which produce neuroinflammation. Along with the accumulation of proteins, neuroinflammation acts as one of the triggers for neuronal damage. In this figure, the role of fatty acids in affecting the important functional pathways of microglia is summarized. Saturated fatty acids (palmitic acid, stearic acid) and omega-6 unsaturated fatty acids (arachidonic acid, linoleic acid) cause inflammatory pathway activation in microglia that is observed through hike in pro-inflammatory cytokine expression (IL-1β, TNF-α) and activation of NF-κB pathway through TLR-4 signaling. Omega-6 fatty acids also tend to increase neuroinflammation, which eventually leads to Tau phosphorylation and its aggregation, whereas omega-3 fatty acids influence phagocytic phenotype in microglia cells in the brain and show anti-inflammatory properties by reducing expression of pro-inflammatory cytokines (IL-1β, TNF-α), NF-κB pathway expression, and increased IKB expression and influence Aβ and Tau phagocytosis in microglia. The ratio of omega-6 to omega-3 fatty acids also decides the occurrence of inflammatory pathway or phagocytic pathway depending upon their dietary ratios

Differential expression of microglial receptors and cytokines decides the type of response in brain environment. In Alzheimer’s disease, extracellular senile plaques and intracellular neurofibrillary tangles are prevalent along with the inflammatory conditions due to activated microglia and astrocytes [53–55]. Therapeutic strategies can be designed to enhance microglia towards phagocytic anti-inflammatory phenotype, which has the capability to clear the debris and other pathogens to reduce the consequences of AD [17, 35, 56, 57]. Out of all organs, the brain has a maximum content of cholesterol as high as 25% of entire body cholesterol. Hence, cholesterol and fatty acids are most important for brain homeostasis as they are an important component of the cell membrane and maintain neuronal plasticity [58, 59]. Dietary fatty acids consumed are reported to cross the blood-brain barrier and hence affect the brain in different aspects [60]. In addition, the presence of fatty acids in myelin sheath also plays an important role in tuning the metabolism of the brain cells mainly by microglia cells. The dietary intake of fatty acids majorly affects the response of microglia cells in the brain. Recent reports suggest that high-fat diet induces inflammatory conditions in the brain mainly in the hypothalamic region [61–63]. Saturated fatty acids increase the proinflammatory phenotype of microglia, whereas unsaturated fatty acids influence the anti-inflammatory phenotype in microglia (Fig. 1). To decrease the adverse inflammatory condition in AD, created by microglia, one of the therapeutic strategies is the supplementation of dietary fatty acids [64]. Fatty acids act as an important metabolic mediator and a rich source of energy; however, excessive consumption of saturated fatty acids generates inflammation in body tissues as well as in the brain [64]. Inflammatory conditions occur due to the accumulation of a large number of macrophages at the sites of infection, which leads to excessive production of inflammatory cytokines. Similarly in the brain, excessive intake of saturated fatty acids (palmitic acid, stearic acid, etc.) promotes the proinflammatory phenotype of microglia [65]. It affects NF-κB pathway, TLR-4 receptors, interferon-γ (IFN-γ), and tumor necrosis factor-α (TNF-α), which are the key factors of inflammatory reaction and therefore control the inflammation in the brain [31]. LPS-mediated activation of NF-κB pathway is a well-known mechanism for inflammatory response [33, 66]. Saturated fatty acids mainly palmitic acid and stearic acids mimic the LPS-induced activation of NF-κB pathway, and reports suggest that this activation is through TLR-4 receptors. Saturated fatty acids act as a ligand for TLR-4 receptors mimicking the LPS and hence activate microglia with increased production of pro-inflammatory cytokines (IL-1β, IL-6, TNF-α), ROS, and NO [31, 32, 67]. Further, this is proved by p65 phosphorylation and translocation into the nucleus, confirming palmitic acid-induced activation of NF-κB pathway (Table 1) [31]. The presence of lipid mediators such as prostaglandins also increases inflammatory reactions, which is catalyzed by cyclooxygenase (COX). Expression of COX at the site of injury is predominant, and its expression depends upon activation of NF-κB cascade, which is observed in LPS as well as with saturated fatty acid-induced microglia [66, 79].

**Table 1:** The classification of fatty acids involved in microglial phagocytosis

Fatty acids	Examples	Function	References
Saturated fatty acids	Palmitic acid (16:0) Stearic acid (18:0)	Elevation of IL-1β, TNF-α TLR-4 signaling pathway Translocation of NF-κB in nucleus	(Tracy, Bergqvist *et al*., 2013 [32]) (Wang, Liu *et al*., 2012 [32])
Omega-3 fatty acids	α-Linolenic acid (18:3n-3) Docosahexanoic acid (22:6n-3) Eicosapentanoic acid (20:5n-3)	Elevation of TGF-β Increase expression of IKB Inhibition of NF-κB Aβ secretion (downregulate amyloidogenic pathway)	(Hjorth, Zhu *et al*., 2013 [53]), (De Smedt-Peyrusse, Sargueil *et al*., 2008 [68]), (Antonietta Ajmone-Cat, Lavinia Salvatori *et al*., 2012 [69]), (Chen, Zhang *et al*., 2014 [70]), (Chen, Wu *et al*., 2017 [71]) (Lim, Calon *et al*., 2005 [72]), (Oksman, Iivonen *et al*., 2006 [73])
Omega-6 fatty acid	Arachidonic acid (20:4n-6) Linoleic acid (18:2n-6)	Tau phosphorylation and aggregation Neuroinflammation	(King, Gamblin *et al*., 2000), (Wilson and Binder 1997 [74])
Omega-6:Omega-3 fatty acids ratio	High omega-6 concentration	Inflammatory response	(Zhu, Wang *et al*., 2016 [75]), (Lafourcade, Larrieu *et al*., 2011 [76]), (Thomazeau, Bosch-Bouju *et al*., 2017 [77]), (Simopoulos 2002 [78])
	High Omega-3 concentration	Anti-inflammatory response	

Unlike saturated fatty acids, polyunsaturated fatty acids having double bonds in carbon chain length have beneficial effects on the brain and eventually reduce neuroinflammation [31, 80]. These polyunsaturated fatty acids are incorporated in the cell membrane as long-chain fatty acids, which help microglia to increase phagocytosis [81]. Long-chain fatty acids increase membrane fluidity and flexibility of the cell membrane. Increase in fluidity alters the expression of receptors on membrane, disrupts receptor signaling, changes lipid raft composition, and affects the curvature of the cell membrane [82]. Reports suggested that polyunsaturated fatty acid-treated microglia express more anti-inflammatory cytokines (TGF-β, IL-10) and other phagocytic markers (CD206, Arg-1, and Ym-1). From the entire class of polyunsaturated fatty acids, omega-3 and omega-6 fatty acids are significant in the brain environment [70, 82, 83]. The influential role of polyunsaturated fatty acids on the brain environment is still needs to be studied (Fig. 1) [53, 83]. Diet-induced inflammation in specific areas of the brain such as the hypothalamus is also a source of excessive activation of Tau phosphorylating kinases [31, 63].

## Role of fatty acids in astrocytes

Fatty acids are important component for development, differentiation, and metabolism in the body [84]. Apart from its role as a component of the cell membrane and nutrients for cells, they also act as signaling molecules in cellular pathways [85]. Astrocytes are the main source of neurotrophic factors and other metabolites that help to maintain neuronal functioning [86]. Fatty acids mainly docosahexaenoic acid (DHA) and arachidonic acid (ARA) are found to release from the cell membrane of astrocytes as a response to various stimuli including inflammation. Released DHA and ARA are then converted into lipid mediators with the help of enzymes COX (cyclooxygenase) and LOX (lipooxygenase) that regulate the inflammatory reaction [75]. Recent studies suggest that astrocytes might play a role in the free fatty acid composition of the brain in response to LPS [87]. Fatty acids are released from the cell membrane of cells depending upon phospholipase enzyme, which are specific for different groups of fatty acids, e.g., for ARA Ca^2+^-dependent enzyme PLA2 and for DHA Ca^2+^-independent enzymes CPLA2, and iPLA2 is responsible [88]. Expression of these enzymes changes the type of response, and conclusive release of DHA and ARA controls the inflammatory phase in CNS [85]. Various pro-inflammatory cytokines secreted by microglia are also known to be produced by astrocytes. Some pro-inflammatory factors, IL-1β, TNF-α, IL-6, and C1q, have the capacity to activate astrocytes leading to neuroinflammation and neuronal death [89]. IL-1β secreted by microglia induces S100β, which is a calcium-binding protein in astrocytes that regulates various events such as proliferation, differentiation and also associated with elevated levels of GSK-3β in the neuron, which is related to Tau pathology [90, 91]. Microglial cytokines regulate the function of astrocytes and also induce it to A1 phenotype that secretes IL-1α, TNF, and C1q. A1 astrocytes lose its ability to stimulate neuronal survival, synaptogenesis, and phagocytosis [92].

## Omega-3 fatty acids influence anti-inflammatory phenotype of microglia

**Fig. 2:**
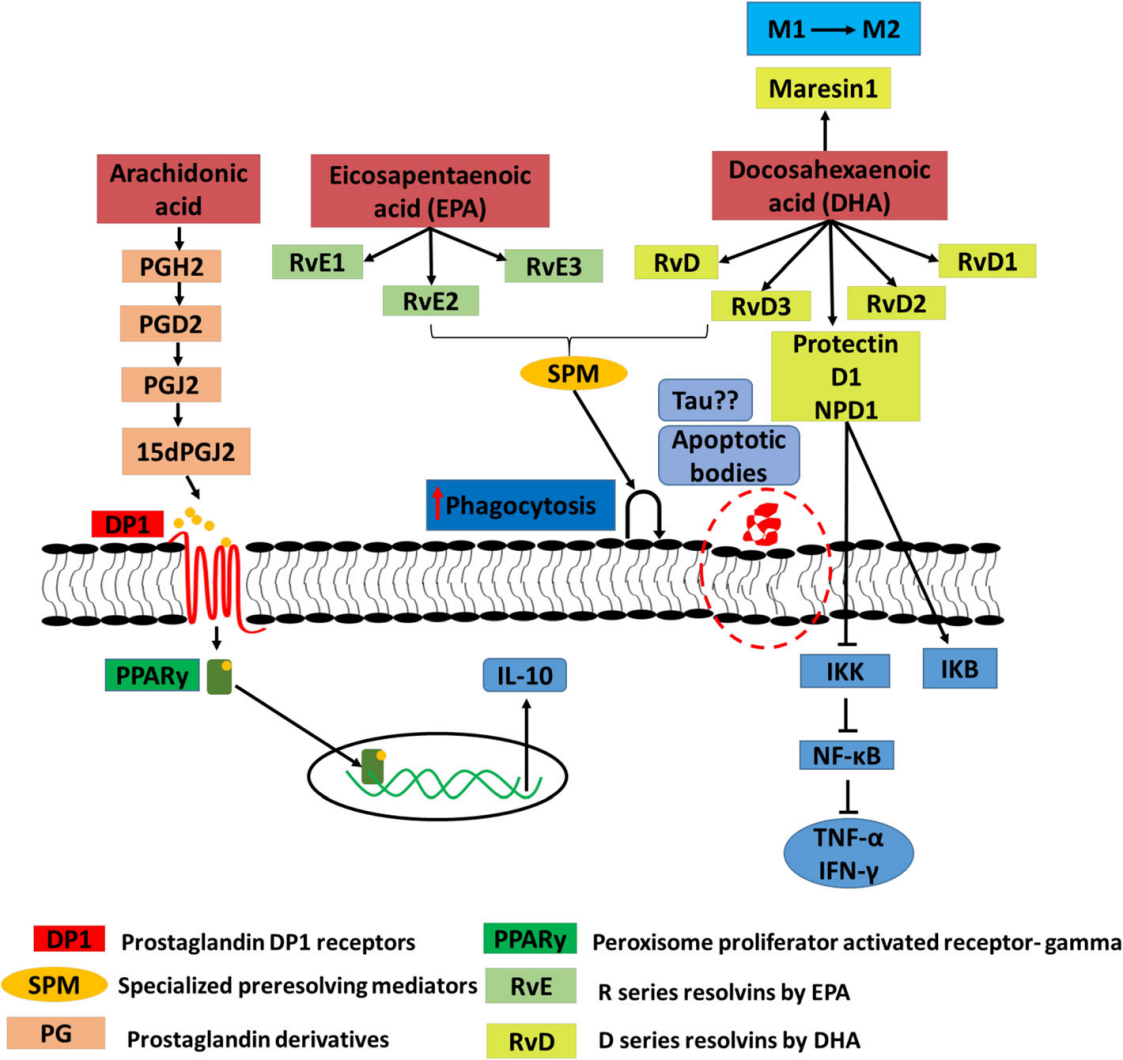
Lipid mediators influencing phagocytosis in microglia. In Alzheimer’s disease, microglia cells majorly drive neuroinflammation. Microglia cells show pro-inflammatory and anti-inflammatory phenotype depending upon the presence of lipid-mediators, receptors expressed, and cytokine secretion. The classical inflammatory phase is marked by the presence of inflammatory cytokines, whereas alternative activation state, i.e., phagocytic state, shows anti-inflammatory state, which drives the clearance of debris, accumulated proteins, etc. Free fatty acids cleaved from cell membrane through action of phospholipase enzymes are targeted to lipid mediator’s synthesis. These lipid mediators then trigger the type of reaction given by microglia cells. Lipid mediators show response depending upon the type of receptors that they bind to microglia cells. PGD2 and PGJ2 stimulate anti-inflammatory response, and they target microglia through DP-1 receptor and exert its anti-inflammatory response through translocation of nuclear receptor PPAR-γ to nucleus increasing production of anti-inflammatory cytokine IL-10, which resolves chronic inflammation; IL-10 has anti-inflammatory property, which is a key regulator of phagocytic phenotype. The E-series (RvE1, RvE2, RvE3) and D-series (RvD, RvD1, RvD2, RvD3) resolvins synthesized from EPA and DHA are together called as SPM (specialized pre-resolving mediators). Protectins (NPD1) suppresses pro-inflammatory response by blocking IKK (activator of NF-κB) and NF-κB and increasing IKB (inhibitor of kappa B). SPM also regulates translocation of NF-κB into nucleus and successive production of inflammatory cytokine (IL-1β, TNF-α); hence, by blocking the NF-κB pathway, SPMs can resolve inflammatory phase efficiently. SPM increases phagocytosis of apoptotic bodies, which includes Aβ protein. Maresin-1, a DHA-derived lipid mediator, which is one of the SPM, has specialty to drive the polarization of microglia from inflammatory to anti-inflammatory phenotype and imparts tissue regeneration

Omega-3 fatty acids contain the first double bond at third and fourth carbon atom in their chain length and considered as essential fatty acids. The omega-3 fatty acids are well known for its neuroprotective function and its importance in neural development in infants and children [53, 81, 93, 94]. Docosahexaenoic acid (DHA-22: 6n-3) and eicosapentaenoic acid (EPA-20: 5n-3) are the most important long-chain fatty acids found in the brain and constitute maximum part of the cell membrane [81, 95, 96]. These fatty acids are incorporated in the cell membrane as long chains of phospholipids. The main source of omega-3 fatty acids is through diet (fish oils), and they can also be synthesized from the precursor molecule α-linolenic acid (ALA) but the rate of conversion is very low as compared to the dietary source [97]. DHA and EPA are preferentially present in cell membrane, myelin, oligodendrocytes, and nerve endings [98]. DHA and EPA are the vital factors to decide the polarity of microglia cells as proinflammatory or anti-inflammatory. DHA tends to increase the anti-inflammatory phenotype of microglia by changing cell membrane composition and influencing the production of lipid mediators. Omega-3 fatty acids also increase the phagocytosis of myelin debris and extracellular Aβ peptide to clear the brain environment in AD, which is the major cause of neuroinflammation (Fig. 2) [53, 70, 73, 99]. The levels of DHA and EPA are found to be drastically reduced with aging in the AD brain; this suggests the vital role of omega-3 fatty acids in the brain [98]. DHA and EPA also have a role in repressing inflammatory cytokines and factors and regulate the process of inflammation. Hjorth et al. showed in CHME3 cell upregulation of phagocytic markers such as CD206 and CD163 and downregulation of M1 markers CD40 and CD86 significantly on treatment with omega-3 fatty acid (DHA) [53, 70, 100]. It has been reported that there is a marked increase in the expression of cellular markers CD163 and CD206 and shows the direct link of DHA to a phagocytic phenotype of microglia [53]. IL-6 production decreased in a concentration-dependent manner in microglia, and there is also increased secretion of neuroprotective factor BDNF. On the basis of proven facts, it can be stated that omega-3 fatty acids are inflammation-resolving fatty acids [53, 82, 101].

Incorporation of long-chain fatty acids into the cell membrane tends to change the lipid composition of the cell membrane and hence, affect the expression of many receptors, activation of downstream signaling molecules, etc. [102]. This also changes the fluidity of lipid bilayer. In AD, Aβ processing is dependent on γ-secretase activity, which is present in the lipid bilayer. Thus, on the treatment of DHA or EPA, Aβ processing changes the non-amyloidogenic pathway [59, 103]. DHA has also been found to activate Akt signaling, increasing neuronal plasticity and cell survival by enhancing the production of BDNF. It also activates Ca^2+^/calmodulin-dependent protein kinase (CaMKII), which is a vital signaling event in the learning and memory process [72]. Aβ-induced activation of Tau phosphorylating kinases like GSK-3β is highly downregulated by DHA and reduces Tau hyperphosphorylation and aggregation [104]. DHA induces PI-3 K/Akt survival signaling and reduces activation of Tau kinases JNK and GSK-3β hence, decreases Aβ and induces pathology of Tau protein [105].

## Omega-6 fatty acids in AD

**Fig. 3:**
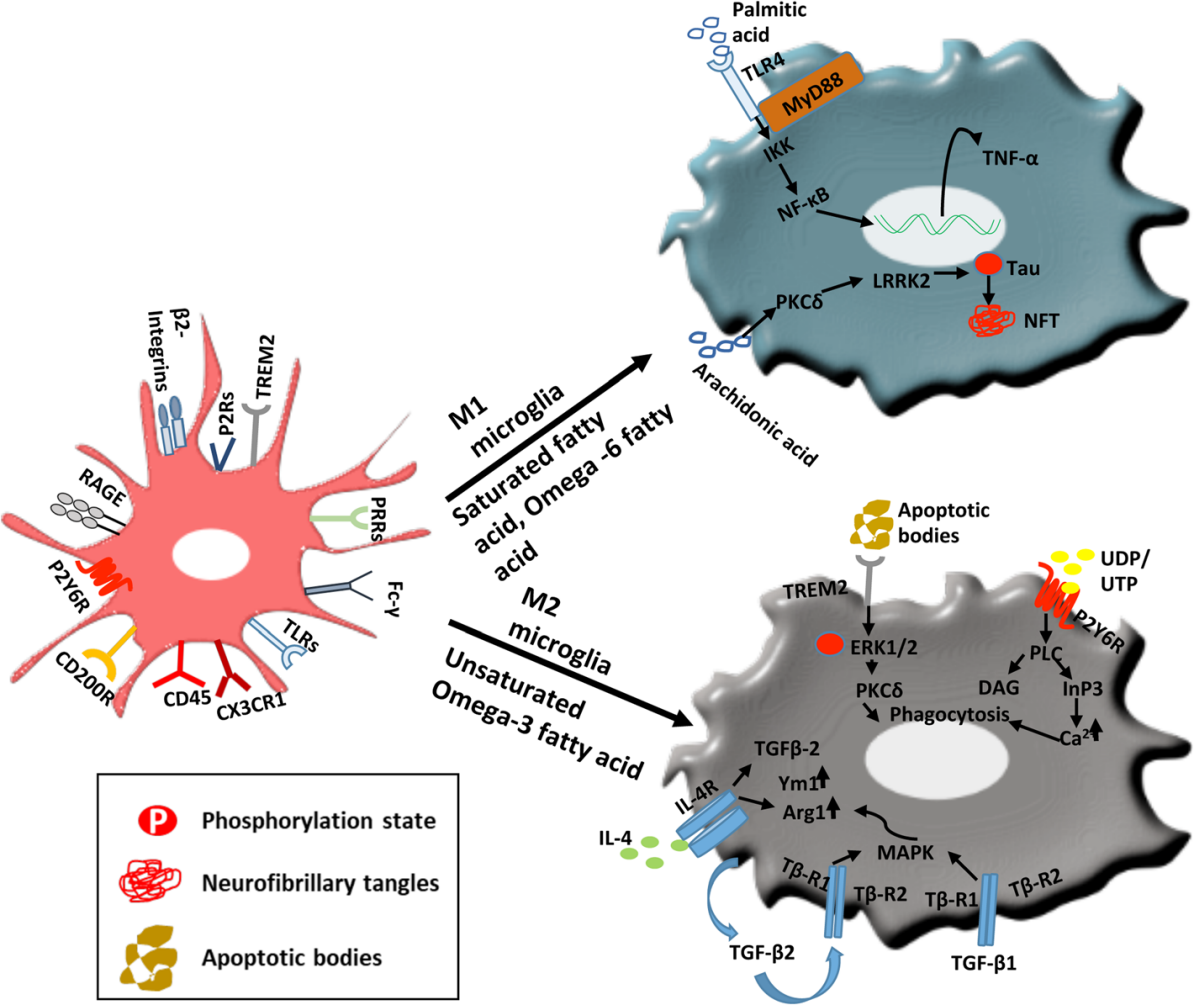
Phenotypic response of microglia on dietary fatty acid exposure. Spreading of Tau between the neurons in tauopathies is a major concern to address that increases the disease progression. The aggregated species of Tau, prudentially oligomer species of misfolded aggregated Tau, show more propagation in the brain environment and found to be more toxic. The phenotype of microglia driven by omega-3 fatty acids could be used as a therapeutic strategy to prevent the propagation of extracellular Tau in tauopathies. Microglia has ramified morphology in surveillant mode, and they significantly express Fc receptors, P2Y6R, β-2 integrins, and PRRs including RAGE and TLRs. Ramified state is maintained through inhibitory signal through interaction of CD200-CD200R, CD22-CD45, and CX3CL1-CX3CR1. Saturated fatty acid (palmitic acid, stearic acid) triggers TLR-4 signaling leading to increase in pro-inflammatory cytokine production (TNF-α); omega-6 fatty acid (arachidonic acid) activating the pathway of PKCδ causes phosphorylation of Tau increasing the aggregation of protein in cells. Thus, it favors inflammatory phenotype and also enhances aggregation of Tau in AD, whereas unsaturated omega-3 fatty acids trigger phagocytic response through TREM2 that recognizes apoptotic bodies and further activates ERK pathway to increase the clearance of apoptotic bodies. P2Y6R receptors interact with UDP released from dead neuron (act as an eat-me signal), which increases phagocytosis of debris, accumulated proteins, and pathogens. This is also indicated by increase in expression of Arginase-1 (Arg-1) and chitinase-like-3 (Ym1) and increases anti-inflammatory cytokine production (TGF-β, IL-4). Along with their anti-inflammatory properties, they also regulate wound healing through Arg-1 and Ym1. Therefore, omega-3 unsaturated fatty acids favor anti-inflammatory phenotype that can mediate clearance of extracellular Tau in tauopathies

In the brain, DHA and ARA are prime components of cell membrane and important precursors for lipid mediators. The brain constitutes 20% of ARA out of total fatty acid content, which is largely present in the form of phospholipids in the cell membrane [106]. DHA preferentially helps to increase memory, synaptic plasticity, learning and decrease inflammation. On the other hand, ARA acts as a mediator for endocannabinoid system, which has an important role in regulating inflammatory responses [107]. The dietary ratios of omega-6 to omega-3 fatty acids play an important role where higher omega-6 to omega-3 fatty acids ratios in diet might affect lipid incorporation in the cell membrane and its consequences in AD [73]. The removal of lipids from the cell membrane is catalyzed by phospholipase A_2_, which is then converted to various lipid mediators by specific enzymes that regulate inflammatory responses. Balance has to be maintained between the enzymes releasing lipids from the cell membrane and the enzymes that incorporate it into cell membrane, which is critical in neuroinflammation [106, 108]. Besides producing lipid mediators, ARA also influences the phosphorylation of Tau protein involved in AD and their aggregation. ARA has been found to activate a number of protein kinases, which have a tendency to phosphorylate Tau protein and mediate their aggregation [109, 110]. Protein kinase C (PKC) are of two types, Ca^2+^-dependent and Ca^2+^-independent; PKC are serine threonine kinase, and their zeta activity is highly enhanced by ARA [109]. Activation of PKCζ leads to phosphorylation of Tau by aiming leucine-rich repeat kinase-2 (LRRK2) protein increasing its aggregation. PKN-α, a member of protein kinase superfamily, was found to be activated by ARA, which tends to phosphorylate Tau protein and also found to be colocalized with aggregated Tau protein. The free fatty acids, ARA, are also known to cause the spontaneous assembly of Tau and amyloid-β protein in vitro even at the sub-micro molar concentration [74]. The inducible and spontaneous assembly of Tau proteins into neurofibrillary tangles is a consequence of a change in the phosphorylation state of protein; the spontaneous assembly of Tau protein in the presence of ARA indicates the same. The enzyme phospholipase A2 (PLA2), which is a Ca^2+^ regulated enzyme, plays an important role coupled with G protein signaling by inducing the cytokines. Level of arachidonic acid is regulated by PLA2; hence, its abundance is a major concern in AD. The spontaneous polymerization of Tau and amyloid-β protein in the presence of ARA indicates the important relevance of PLA2 in AD (Fig. 3) [74].

## Lipid mediators drive microglial phenotype

One of the major causes of neuroinflammation is excessive production of lipid mediators. The synthesis of lipid mediators proceeds in three stages: pre-inflammatory to pro-resolution phase followed by the final anti-inflammatory phase [111]. Fatty acids are released from the cell membrane after the action of an enzyme such as phospholipase A_2_, which is a cytosolic enzyme activated by Ca^2+^ increase and by MAP kinase after its phosphorylation at Ser^505^ residue [112]. The release of ARA and DHA from the cell membrane is well-studied mechanisms; these free fatty acids are then used for the synthesis of lipid mediators, which mediate inflammatory reaction. The conversion into lipid mediators is carried out by majorly three enzymes, cyclooxygenase (COX), lipooxygenase (LOX), and cytochrome P450, where COX-1/2 expression decides the type of response [81]. The COX-1 enzyme is constitutively expressed in most of the body tissues while COX-2 enzymes overexpress only in inflammatory response; hence, it is considered as a marker for inflammation. The inhibition of COX-1/2 is a crucial therapeutic mechanism to reduce inflammation. Nonsteroidal anti-inflammatory drugs (NSAIDs) inhibit COX-1/2 and act as an important treatment for AD to reduce the inflammation. In AD brain, an elevated level of COX-1 expression by microglia indicates the inflammatory response, which is also observed in Aβ treatment in microglia cells [106]. Inhibiting COX-2 and LOX-5 proved to inhibit the inflammatory pathway by blocking the effect of NF-κB, with increased IL-10 concentration but decreasing the TNF-α and IL-6 expression in AD [113]. ARA-derived lipid mediators have varied effects as it gives rise to the production of many prostaglandins (PGs) and leukotrienes (Lxs). ARA produce vital signaling molecule prostaglandin E2 (PGE2) and prostaglandin D2 (PGD2), which are synthesized from PGH2 by the enzymes PGE2 and PGD2 synthase, respectively, and their presence is abundant in glia cells mainly in microglia. PGE2 has an anti-inflammatory effect, which helps to resolve the inflammation and also helps to clear debris and accumulated proteins by phagocytosis. It is also found to increase IL-10 expression, which helps in resolving the inflammatory phase [108]. Overexpression of PGE2 during initial stages of inflammation indirectly increases the transcription of genes required for lipid mediators such as maresin (MaR), resolvins (Rv), and protectin (PD) PGD2, which adds to its anti-inflammatory effect by binding to DP1 receptor and through peroxisome proliferator-activated receptor (PPAR-γ); a nuclear receptor is involved in increased production of IL-10. In this pathway, PGE2 first gets converted to its biologically activated form of PGJ2 by non-enzymatic dehydration. It also inhibits IKB kinase (IKK) directly, which is an activator of NF-κB signaling and initiates tissue repair and remodeling [113, 114]. Specialized pro-resolving mediators (SPMs), D series resolvins and E series resolvins (Rv), protectins (PD), and maresin (MaR) are synthesized by LOX pathway from DHA and EPA. RvD, MaR, and PD are synthesized by DHA, whereas RvE is from EPA (Fig. 2) [115]. SPMs bind to cellular receptors for their functioning, e.g., RvD1 acts via binding to GPR32, RvE1 through ChemR23 orphan receptor [116]. SPM increases phagocytosis and suppresses inflammation by blocking the NF-κB pathway and PPAR-γ-mediated mechanism. According to lipid profiling, anti-inflammatory phenotype of microglia produces more quantity of protectins, maresin, and D-resolvins, whereas inflammatory phenotype of microglia produces more of ARA-derived proinflammatory markers [117, 118]. Recently, the MaR1 pathway is found effective in the resolution phase. MaR1 is primarily synthesized in the inflammatory phase, and their concentration increases in the subsequent resolution phase. MaR1 along with RvE1 initiates tissue remodeling. SPMs competitively bind to COX-2 over PGs and initiate resolution phase. Therefore, an increase in dietary ratio of n-3 fatty acids to n-6 fatty acids provides a beneficial role in the reduction of inflammatory response. Lipid mediators, therefore, play an important role in phenotype remodeling in microglial cells and efficiently support the switching of pro-inflammatory to anti-inflammatory phenotype of microglia. They also enhance the ability of anti-inflammatory cells to undergo phagocytosis where IL-10 plays a key role in the tuning function (Fig. 2) [113].

## Fatty acids influence differential cytokine production in microglia

The cytokine profile of microglia depends upon the phenotype of microglia. The polarization of microglia is a significant process, which decides the fate of reaction. The polarization of microglia can be monitored by plasma fatty acid level, and it alters with respect to various dietary habits. Since fatty acids can cross the blood-brain barrier through simple diffusion, their level in the plasma directly affects microglia cells. Therefore, gliosis also arises due to obesity, which triggers metabolic dysfunction [17, 35].

Different classes of fatty acids drive the polarization of microglial cells by deciding the flexibility of the cell membrane, which in turn formulates the type of receptors and a response given by cell. Long-chain saturated fatty acids polarize microglia towards the inflammatory phenotype by increasing the level of cytokine secretion critically of TNF-α, IL-6, and IL-1β [31]. The secretion of these pro-inflammatory cytokines has been observed in cultured astrocytes in a dose-dependent manner [119]. Palmitic acid (PA) is the most potent candidate of all the saturated fatty acid class to cause increased expression of pro-inflammatory cytokines followed by stearic acid and lauric acid. Omega-6 fatty acids including ARA and linoleic acid also show increased expression of the pro-inflammatory cytokine on treatment, but the levels are less as compared to palmitic acid in astrocytes [119]. In microglial cells, PA induces marked upregulation of IL-1β, which is a key mediator of inflammatory response. IL-1 overexpression by microglia cells ensures the upregulation of IL-1β, IL-6, and microglia activation. The cytokine profile favors neuronal stress and neurodegeneration. NF-κB-mediated inflammatory responses have been observed due to the activation of TLR-4 pathway by palmitic acid. Saturated fatty acids showed its effect on TLR-4 activity by inducing changes in lipid rafts and hence activating downstream NF-κB inflammatory reaction. IL-6 production, on the other hand, is totally independent of NF-κB pathway and is not affected by omega-3 fatty acids (Fig. 3) [31].

The condition of neuroinflammation arose due to the fact that the balance between the pro-inflammatory phase and the anti-inflammatory phase is not properly maintained. Microglia could be a direct target to address, which can decrease the degree of disease. Omega-3 fatty acids especially DHA have the ability to reduce the pro-inflammatory cytokines secreted by microglia [35, 69]. Omega-3 fatty acids and their products such as neuroprotectins (PD) and resolvins (Rv) have anti-inflammatory properties. DHA has been found to inhibit the significant inflammatory pathway NF-κB and activates pathways, which modify the LPS, and IFN-γ induced a response. DHA also influence phosphorylation of P38 MAPK kinase, which is most important in inflammatory cytokine production and increases nuclear translocation of PPAR γ, which results in the production of anti-inflammatory cytokine IL-10. DHA also restores the level of IGF-1 that is reduced in LPS-induced reactions [120]. In conclusion, omega-3 fatty acids reduce microglial activation and aid anti-inflammatory phenotype [121].

## Spreading of Tau influenced by microglia

**Fig. 4:**
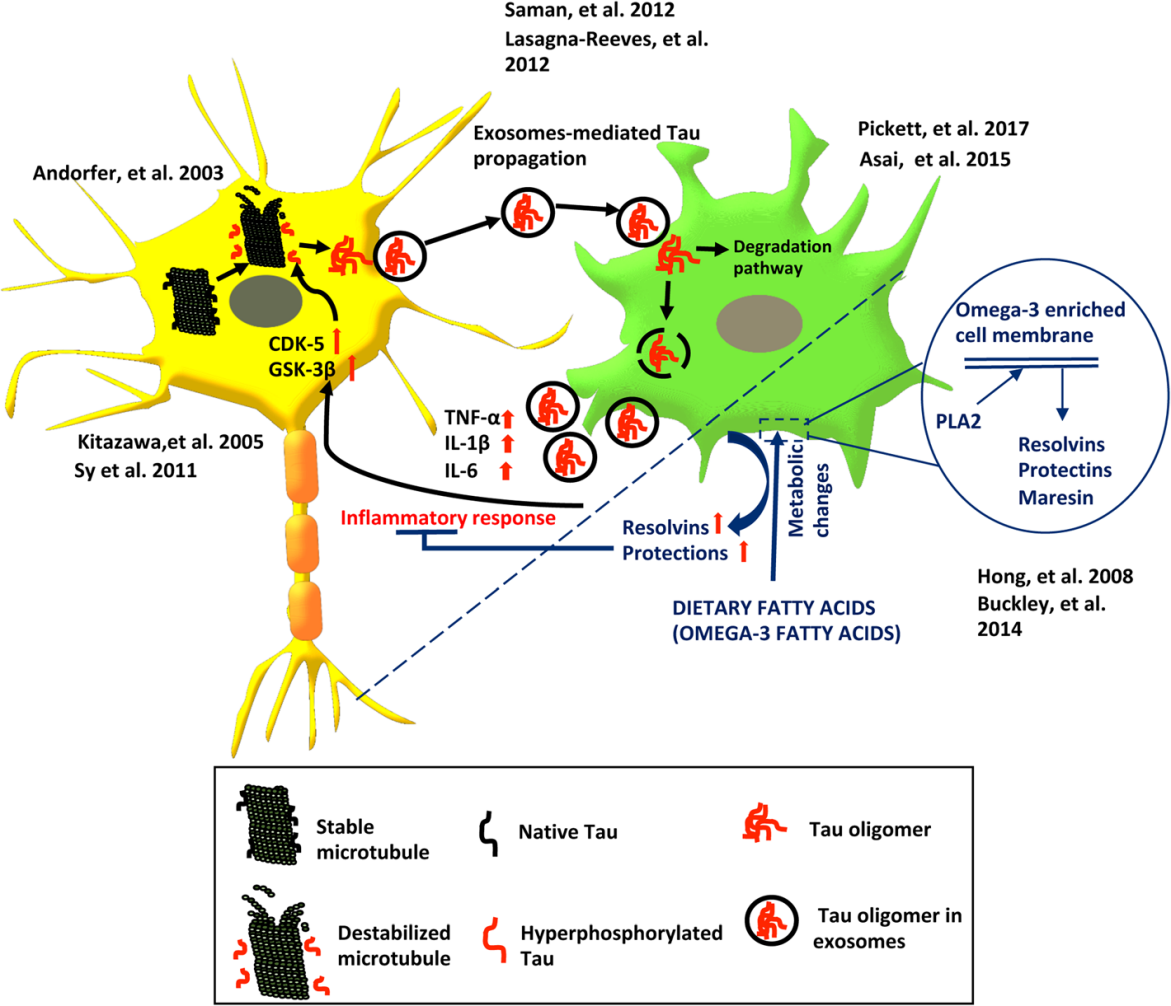
Propagation of Tau mediated by microglia. Tau is a microtubule-associated protein, stabilizes microtubule, and supports cargo transport. On abnormal hyperphosphorylation, Tau protein detaches from the microtubule and aggregates in the form of oligomers to further accumulate in the form of neurofibrillary tangles inside the neuron. The aggregated Tau protein, especially oligomers, has the tendency to spread within the neuron and cause template-dependent misfolding of normal Tau protein which is called seeding effect of Tau. One of the mechanisms for the spreading of Tau is exosome-mediated spreading. Exosomes are further taken up by microglia, which can either undergo degradation pathway or can be re-released in the brain environment enhancing Tau propagation. Due to the presence of aggregated Tau protein, microglia become activated and influence the inflammatory response through cytokine secretion (IL-1β, IL-6, TNF-α). The inflammatory cytokines enhance Tau pathology by reciprocating enhancement of expression of phosphorylation-dependent kinases CDK5 and GSK-3β in neuron increasing Tau hyperphosphorylation. Dietary omega-3 fatty acids repress the inflammatory response by enhancing the synthesis of lipid mediator’s resolvins and protectins by microglia. We hypothesize that the lipid mediators can resolve the inflammatory phase and hence can reduce Tau pathology by decreasing expression of Tau phosphorylating kinases

Unusual accumulation of Tau protein in the form of neurofibrillary tangles is one of the reasons of neuroinflammation. Aberrant gliosis is, therefore, an indirect measure of accelerated Tau pathology and successive neurodegeneration. Secreted inflammatory cytokines have proved to activate astrocytes in the vicinity [122]. The excessive glial activation has the ability to modify Tau pathology by activating kinases, which have an effect on driving pathological Tau [123]. In normal conditions, Tau is bound to microtubule filaments and regulates its stability and assembly and also facilitates cargo transport through microtubules [124–126]. In order to remain bound to microtubule filaments, there is phosphorylation at serine threonine residues, which also regulates its conformational changes. Any abnormal phosphorylation alters Tau conformation, which results in detachment from microtubules [127, 128]. The free hyperphosphorylated Tau has a greater tendency to acquire beta-sheet conformation and accumulate as neurofibrillary tangles [124]. A neurofibrillary tangle gets deposited in neurons and leads to neuronal loss. With the existing data, it has been proved that these pathological Tau species spread within the neuronal network and cause propagation of Tau [38, 129–132]. The released Tau has been detected in CSF and also has the ability to be taken up by neighboring cells through various mechanisms [133–135]. After getting internalized into the cell, the abnormal aggregated Tau has the ability to induce template misfolding that proceeds to seeding effect of Tau [45, 136, 137]. Microglial activation on the other side acts as a positive regulator of Tau as secreted cytokines drive the hyperphosphorylation of Tau and its aggregation [123]. However, microglia also contributes to Tau spreading; they take up both soluble and insoluble aggregated forms of Tau. Internalized Tau can undergo by two pathways, either they undergo degradation or there is re-release of Tau species outside the cell [138–140]. The discharge of Tau is in the form of exosomes through various mechanisms [135, 141]. Microglia has the capacity to phagocytose Tau and re-released in exosomes, which would cause the spreading of Tau. Reduction in exosome synthesis can reduce Tau spreading [142]. The inflammatory response given by microglia alters Tau, independent of exosomes-mediated mechanisms. It has been proved that dietary intake of fatty acids could be effective to prevent AD and can act as an additional therapy for AD (Fig. 4) [102]. Dietary fatty acids especially omega-3 fatty acids mediate anti-inflammatory response in microglia by affecting the production of lipid mediators such as resolvins and protectins [113] Omega-3 fatty acids therefore can be used as therapeutic strategies to reduce microglial activation that would control Tau pathology. Here, we suggest that by reducing the inflammatory response, Tau pathology can be reduced. The level to which gliosis can be controlled through dietary fatty acids especially omega-3 fatty acids is an area of research to contribute.

## Conclusions

In this study, we have focused on the role of dietary fatty acids in tuning the microglial polarization. In AD-like neurodegenerative disease, inflammatory microglia is more predominant due to the accumulation of abnormal proteins. Inflammatory phenotype of microglia lacks the clearance ability, which eventually leads to excessive accumulation of protein such as Aβ and Tau. According to the literature, dietary fatty acids especially omega-3 fatty acids have the ability to affect the brain environment to a great extent, as it constitutes most of the fatty acid content of the brain. Omega-3 fatty acids, hence, can enhance the polarization of microglia towards anti-inflammatory phagocytic phenotype by upregulating or downregulating expression of certain receptors on the surface of microglia changing its phenotype. They also affect the flexibility of the cell membrane that contributes to the polarization state of microglia. Of interest, the ability of microglia can be actually taken under consideration to clear the accumulated protein in AD, which is the major concern. Propagation of Tau protein, oligomer species, and aggregated proteins, which are released outside the cells, could be engulfed by anti-inflammatory phagocytic phenotype of microglia, and their clearance will eventually reduce the propagation in Tauopathies like AD. Extracellular Tau burden induces template-dependent aggregation of healthy Tau and hence, the spreading of disease. Dietary fatty acids play a major role in increasing the percentage of anti-inflammatory phenotype of microglia that ultimately dominates the environment and can be used as a potential therapeutic tool to reduce the burden of accumulated protein. Another aspect is that omega-3 fatty acids act as a competitive inhibitor of ARA to reduce the production of inflammatory lipid mediators’ production. Omega-3 fatty acids hence reduce the inflammatory condition. The overall effect of omega-3 fatty acids would help the brain environment to improve and reduce pathological conditions of AD.

## Data Availability

This review does not contain any analyzable data. All authors cited in this paper are publicly available.

## Abbreviations

AD: Alzheimer’s disease
ARA: Arachidonic acid
CaMKII: Ca^2+^/calmodulin-dependent protein kinase
COX: Cyclooxygenase
DAM: Disease-associated microglia
DHA: Docosahexanoic acid
EPA: Eicosapentanoic acid
HD: Huntington’s disease
IFN-γ: Interferon-γ
IKK: IKB kinase
LOX: Lipooxygenase
LRRK2: Leucine-rich repeat kinase 2
MaR: Maresin
NFTs: Neurofibrillary tangles
NO: Nitric oxide
NSAIDs: Nonsteroidal anti-inflammatory drug
PD: Parkinson’s disease
PD: Protectin
PG: Prostaglandins
PHF: Paired helical filaments
PKC: Protein kinase C
PLA2: Phospholipase A2
ROS: Reactive oxygen species
Rv: Resolvins
SPM: Specialized pro-resolving mediators
